# Assessing Yield and Quality of Melon (*Cucumis melo* L.) Improved by Biodegradable Mulching Film

**DOI:** 10.3390/plants12010219

**Published:** 2023-01-03

**Authors:** Eugenio Cozzolino, Ida Di Mola, Lucia Ottaiano, Maurizio Bilotto, Milena Petriccione, Elvira Ferrara, Mauro Mori, Luigi Morra

**Affiliations:** 1 Council for Agricultural Research and Economics (CREA)—Research Center for Cereal and Industrial Crops, 81100 Caserta, Italy; 2 Department of Agricultural Sciences, University of Naples Federico II, 80055 Portici, Italy; 3 Council for Agricultural Research and Economics (CREA)—Research Center for Olive, Fruits and Citrus Crops, 81100 Caserta, Italy; 4 Department of Environmental Biological and Pharmaceutical Sciences and Technologies, University of Campania “Luigi Vanvitelli”, 81100 Caserta, Italy

**Keywords:** biodegradable mulching film, muskmelon, yield, fruit quality, bioactive compounds, total soluble sugars, flesh color

## Abstract

Low-density polyethylene (LDPE) plastic mulching films have an important function, but at the end of their lifetime pose an economic and environmental problem in terms of their removal and disposal. Biodegradable mulching films represent an alternative to LDPE with the potential to avoid these environmental issues. In this preliminary study, we employed a biodegradable film based on Mater-Bi^®^ (MB) in comparison with low-density polyethylene to assess their effect on the yield and particular quality traits (organoleptic and nutraceutical composition of the fruits) of muskmelon (cv Pregiato) grown on soils with different textures (clay–loam—CL and sandy loam—SL) in two private farms in South Italy. Soil temperature under the mulch was also measured. During the monitored periods, mean soil temperature under LDPE was higher (about 1.3 °C) than that under the biodegradable film and was higher in SL soil than in CL soil, at 25.5° and 24.2 °C, respectively. However, the biodegradable film was able to limit the daily temperature fluctuation, which was 1.7 °C in both soils compared with 2.3 °C recorded for LDPE. Fruit yields were higher with MB film than LDPE (+9.5%), irrespective of soil texture. MaterBi^®^ also elicited increases in total soluble solids, polyphenols, flavonoids, and antioxidant activity compared with LDPE films: 13.3%, 22.4%, 27.2%, and 24.6%, respectively. Color parameters of flesh, namely brightness, chroma, and hue angle were better in fruits grown on LDPE. Our findings suggest that Mater-Bi^®^ based biodegradable mulching film is a potentially valid alternative to traditional LDPE, particularly for obtaining the agronomical benefits outlined above and for promoting environmental sustainability due to its favourable biodegradable properties.

## 1. Introduction

Mulching has increasingly been used due to its well-known beneficial effects which include increasing soil temperature [1] and moisture, reducing nutrient losses [2], and inhibiting growth of weeds [3,4,5], thereby enhancing plant growth and yields [6,7,8].

Among the most frequently used materials for mulching, plastic films play a key role; they are usually manufactured using low-density polyethylene (LDPE) and their life cycle in the field depends on many factors, including climate. At the end of their lifetime, plastic films must be removed from the field and disposed of according to the European directives relevant to waste management (99/31 EC, 2000/76 EC, 2008/98/EC). However, these plastic films are not always suitably disposed of and, consequently, plastic residues may pollute the soil. Yang et al. (2020) [9] reviewed the progress of various renewable materials used for biodegradable mulch; these renewable resources contain mainly starch, cellulose, poly-lactic acid (PLA) and polyhydroxyalkanoate (PHA). The biodegradable mulch films are produced by biodegradable polymers, defined as polymers obtained from renewable resources, along with fossil-based biodegradable polymers [10]. Recent studies have demonstrated that repeated application of different bio-based biodegradable films onto soil at the end of the crop cycle does not deleteriously modify the soil’s physical properties, health indicators, and functions [11]. Paper mulch degrades completely into the soil in less than 12 months, while Mater-Bi^®^ (NOVAMONT) and Ecovio^®^ (BASF) biodegrade completely but more slowly depending on climate, soil texture, and biological activity [12]. Razza et al. [13] effectively adapted the calculation of the material circularity indicator, well established for traditional plastic products, and applied it to bio-based and biodegradable mulch films with the aim of demonstrating the viability of these products for use in a circular economy. The market for biodegradable mulch film is expected to grow at a rate of 8.5% during the forecast period 2021–2026, according to the report by Mordor Intelligence (2022) [14]. The European market for mulch films exceeds 100,000 metric tons per year, and the global market exceeds 2 million tons annually. Supportive regulation from governments across the world to promote organic mulching is driving the market. For instance, in Europe, EN 17033 is the most recent product standard for biodegradable mulch films used in agriculture and horticulture, and specifies the relevant requirements and test methods [15]. The standard is designed to provide a clear reference for farmers, distributors, and stakeholders. In recent years, considerable research has been conducted into the applicability of biodegradable mulching films for horticultural crop cultivation, including lettuce [16,17], zucchini [18], strawberry [19], tomato [20], melon [21], and many other vegetables [5,22], often eliciting yields higher than or at least comparable to those obtained using traditional plastic films.

Melons and other cucurbit crops benefit from mulching in an open field, with tunnels and greenhouses useful for obtaining early yields. The ability of mulching materials to hasten the crop cycle depends mainly on their transmissivity of solar and long IR radiation. Vox et al. [23] reported that transmissivity in the long IR electromagnetic spectrum of 20 μ Mater-Bi (MB) black film is about half of that of 40 μ LDPE film, despite being half the thickness. That is to say, the potential for black MB mulch to maintain soil heat and induce early growth is higher than for LDPE, provided the mulch film remains intact. Starting in 2003, Candido et al. [24] carried out several trials to test the influence of Mater-Bi mulch films on the yields of melon, the earliness of cropping, and control of weeds. Clear, black, and green colored films were tested in successive years. Trials with melon in open fields in south-east Spain and with winter melon in Sicily demonstrated that clear MB produced similar yields to LDPE in terms of quantity and quality, and these results were better than achieved using black MB of equal thickness (15–18 μm) [25,26,27]. Waterer et al. [22], in experiments carried out in Canada in 2007 and 2008 with cantaloupe melon, detected no differences in productivity between clear and black biodegradable mulches. Clear mulch films require the use of pre-emergence herbicides for controlling weeds that are favored by the light, warm, and humid conditions under the film [5]. Therefore, MB green mulches were introduced to provide a better combination of soil warming and weed control. Yields and quality were similar to those of black MB and LDPE, but film degradation was faster [28]. Filippi et al. [29] found that yields as well as the content of soluble solids (°Brix) with green mulch were higher than LDPE. More recently, Cozzolino et al. [30] assessed a new grade (EF04P) of 15 μ black MB film, in comparison with 50 μm black PE film, under a tunnel during the spring–summer cycle. They found no differences in terms of marketable yields, mean weight of fruit, or hardness of flesh, while fruits grown on MB mulch presented higher values of total soluble solids, carotenoids, flavonoids, and antioxidant activity than those grown on LDPE.

The aim of the current research was to assess the agronomical response to biodegradable mulching, compared with traditional polyethylene, in a cultivar of muskmelon grown on two soils with different textures. This study also evaluates the effect of the two experimental factors on the organoleptic and nutritional qualities of fruits.

## 2. Results

### 2.1. Soil Temperature under Mulching

**Figure 1:**
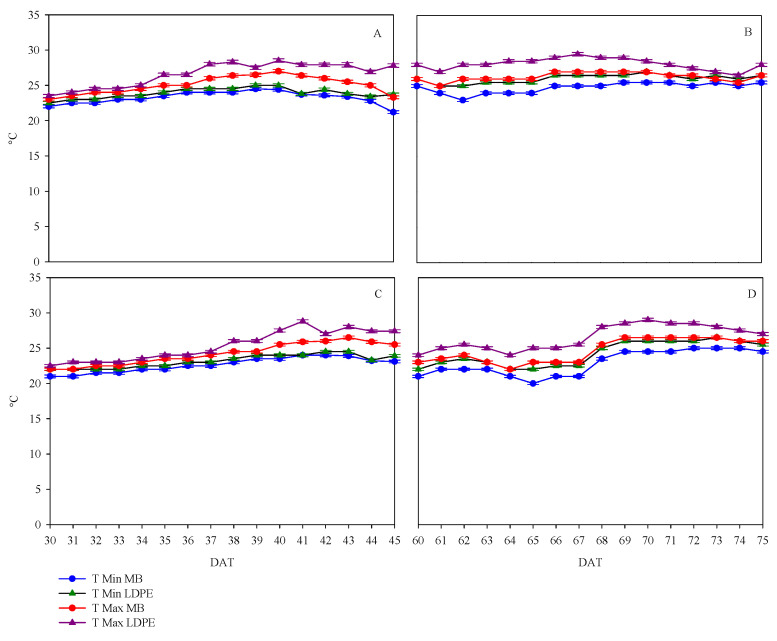
Average daily trends of maximum and minimum temperatures under the two mulching films (biodegradable film—MB; low density polyethylene—LDPE) in sandy loam soil (SL—Pellegrino farm) and clay–loam soil (CL—Ferrara farm) during two periods of muskmelon cycle (**A** = 30–45 DAT, **B** = 60–75 DAT for SL; **C** = 30–45 DAT, **D** = 60–75 DAT for CL). Vertical bars show the standard error.

The average daily trend of maximum and minimum temperature under mulching is reported in Figure 1, for sandy loam (SL) soil (Figure 1A,B) and clay–loam (CL) soil (Figure 1C,D). Specifically, the soil temperature in both farms was monitored over two periods: from 30 to 45 days after transplant (DAT) and from 60 to 75 DAT.

Interestingly, the temperature (mean value of the two periods) of sandy loam soil was always higher than that of clay–loam soil, 25.5 °C vs. 24.2 °C, respectively (Figure 1A–D). In addition, the temperatures in both soils under LDPE were higher than those under biodegradable film; in the sandy soil, the mean values of the two periods were 26.2 °C for LDPE and 24.9 °C for MB (Figure 1A,B), while in the clay–loam soil, the mean values were 24.8 °C and 23.6 °C, respectively (Figure 1C,D). In SL soil, the temperature differences between the two mulching systems were more evident, starting from 35 DAT and reducing after 70 DAT (Figure 1A,B). In CL soil, the difference between the temperatures started at 38 DAT but the temperatures remained approximately constant until the end of the second period (Figure 1C,D). Finally, the biodegradable film was able to limit the daily temperature excursion, which was 1.7 °C in both soils against 2.3 °C recorded for LDPE as the mean of the two periods for both soils (Figure 1A–D).

### 2.2. Yield as Affected by the Soil Texture and Mulching

**Figure 2:**
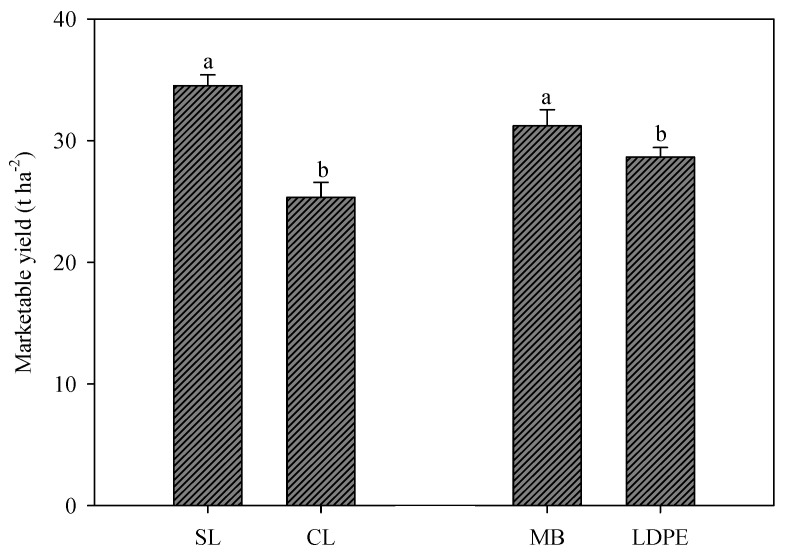
Marketable yields of muskmelon affected by soil texture (clay–loam—CL; sandy loam—SL) and mulching (biodegradable film—MB; polyethylene—PE). The vertical bars show the standard error; different letters indicate statistical differences.

The total marketable yield (sum of all harvests) of muskmelon was significantly affected by the soil texture and mulching. On the sandy soil (Pellegrino farm), the yield reached almost 35.0 t ha^−1^, about +36.3% more than the yield recorded on clay–loam soil (Figure 2).

The biodegradable mulching elicited a higher yield than PE, about +9.1% (Figure 2).

The quantity of marketable fruits and their average weight were affected by soil texture only, while the percentage of unmarketable to marketable yield was affected by the interaction between the two experimental factors (Table 1). Notably, the sandy loam soil elicited an increase in the number and average weight of marketable fruits, +43.8%, and +7.8%, respectively (Table 1). The highest values of unmarketable yield were recorded for plants grown on clay–loam covered by the biodegradable mulching film, +66.1% higher than the mean value of all other treatments; the best performance (lowest value of unmarketable yield) was recorded in plants grown on sandy loam soil covered by LDPE; no differences were found between CL-LDPE and SL-MB (Table 1). Regarding the percentage incidence of unmarketable to marketable yield, this followed a slightly different trend and the highest values were recorded in CL-MB, almost double the mean value of all other treatments, among which these values did not greatly differ (Table 1).

The analyzed parameters of the nutritional quality of muskmelon fruits were differently affected by the experimental factors. The interaction was never significant; polyphenols and antioxidant activity (AA) were statistically affected by both factors. Flavonoids were only affected by mulching, and carotenoids only by soil texture (Table 2).

Notably, the biodegradable mulching enhanced the quality of fruits and contributed to 27.2%, 22.4%, and 24.6% increases for flavonoids, polyphenols, and AA, respectively (Table 2). Interestingly, the sandy loam soil elicited an improvement in certain quality traits: 14.1%, 10.0%, and 22.8% increases for polyphenols, carotenoids, and antioxidant activity, respectively (Table 2).

The pH and titratable acidity were not affected by the experimental factors, and showed mean values of 6.71, and 2.085, respectively (Table 3). The firmness and dry matter percentage of muskmelon fruits were affected only by soil texture, with a higher value of the dry matter in clay–loam soil and a lower value for firmness (Table 3). Finally, the total soluble solids were affected by both experimental factors, with an 8.1% increase in clay–loam soil compared with sandy loam soil, and a 13.3% increase in MB compared with DPE (Table 3).

Table 4 reports the effect of interaction between Soil texture × Mulching on L* and h, and the main effect of the two factors on C. Notably, the flesh of the fruit from plants grown on clay–loam soil covered by LDPE showed the highest L* values, significantly different from all other treatments (+10.4% compared to the mean value of the other three treatments). A similar trend was recorded for the h value, with a lower increase (3.8%). For the C parameter, the highest value was recorded in sandy loam soil (41.4 vs. 38.6 of CL), and for the mulching, LDPE elicited higher values (43.01 vs. 36.97).

## 3. Discussion

In the recent years, environmental sustainability has become an imperative goal of modern agriculture. Given that polyethylene mulch films need 100 years to degrade [31] and their disposal is difficult and/or expensive, it is essential to find a sustainable alternative such as biodegradable film.

Considerable research has been conducted on the effect of biodegradable films on the growth and yields of several food crops [16,17,18,32,33], but few studies in this context have assessed food quality and how it is affected by the use of biodegradable films.

In the current research, we evaluated the effects of a biodegradable film compared with polyethylene film on the yield and certain quality traits of muskmelon cultivated on two soils with different textures (clay–loam and sandy loam).

In contrast to the study by Vox et al. [23], LDPE promoted a higher temperature than black biodegradable MB film in the topsoil layer, with about a 7.9% increase in the mean value compared with reports from previous research [16,29]). Lopez-Marin et al. [34] recorded similar behavior using polyethylene compared with biodegradable film; Moreno et al. [35] suggested that this behavior may be due to the composition of the biodegradable film, which permits increasing gas exchange with the open air as a result of its higher permeability to water vapor. Notably, the biodegradable film reduced the temperature variations between day and night, with daily fluctuations of 1.7 °C and 2.3 °C for MB and LDPE, respectively. This reduced fluctuation in daily temperature may contribute to limiting abiotic stress in roots, even though the soil temperature in SL and CL ranged between 20–27 °C, optimal levels for vegetative and reproductive growth in melons [36,37]. Overall, the mean temperature in sandy loam soil was about +5.4% higher than that recorded in clay–loam soil (25.5 vs. 24.3 °C, respectively). The MaterBi^®^ film elicited higher yield (+9.5%) compared with the traditional mulching film (LDPE), which may be attributable to a slightly higher number of fruits per plant, although this increase was not significant (see Table 1). In a previous test using melon, Wang et al. [38] compared two different biodegradable films with polyethylene and bare soil. They recorded higher yields on biodegradable film, mainly due to higher fruit weight. Furthermore, Filippi et al. [29] found that melon plants grown on biodegradable mulching film produced more fruits per plant irrespective of the film color, but without significant differences in yield. In a greenhouse test using melon plants grown on biodegradable film, Candido et al. [24] obtained similar yields to those using clear PE. In summary, it is well-established in the available literature that MB mulch does not reduce yields of melon compared with LDPE mulch [22,25,26,27,28]. We assume that the higher productivity demonstrated by plants grown on MaterBi^®^ could be attributable to higher nitric nitrogen availability. Biodegradable films have higher breathability than LDPE films, therefore the humidity is often greater under the latter, creating anaerobic conditions which favor the denitrification process [39] and reduce the nitrate content available for plants. Among qualitative traits, TSS content is an important parameter because it is positively correlated to the sweetness of the pulp and, therefore, to the acceptability of the product for consumers. Indeed, the literature reports that consumer preferences for melon mainly depend on parameters including sweetness, aroma, consistency of pulp, and, more recently, phytonutrient content [40,41].

With regard to the influence of mulching on fruit quality, we recorded a significant 13.3% increase in TSS of fruits from plants grown on MB mulch in both the sandy and clay–loam soils. Wang et al. [38] reported higher levels of soluble sugars in fruits grown on biodegradable films based on poly (butylene adipate-co-terephthalate)/polylactic acid/lignin compared with polyethylene.

Interestingly, we found that the MB biodegradable film substantially improved other traits of fruit quality. In particular, it boosted flavonoids, polyphenols, and antioxidant activity (AA) by 27.2%, 22.4%, and 24.6%, respectively. While this had not previously been reported in the literature relating to muskmelon, similar results were reported in strawberries [42] and partially confirmed by Morra et al. [43].

Recent studies have demonstrated that these compounds play an important nutritional role. They are absorbed during digestion and play numerous regulatory roles in cellular metabolism [44]. Melon fruit contains polyphenols, a group of phytochemical compounds that have antioxidant, antimutagenic, antiproliferative, and antimicrobial capacities, and are involved in the prevention of and response to several pathologies. Furthermore, the fruit is rich in carotenoids (α-carotene, β-carotene, p-cryptoxanthin, lutein, and zeaxanthin), which are responsible for the orange coloring of the pulp and provide a natural source of antioxidants.

Several researchers have reported that the effect of mulching on the organoleptic and nutraceutical characteristics of fruit depends on the typology of the film [45,46]. However, studies have reported results that contrast with our current findings, having shown that the use of non-degradable plastic mulching films improved the content of soluble solids and the total content of flavonoids, polyphenols, and anthocyanins in fruits of different species, such as vine (*Vitis vinifera* L.) [47], strawberry (*Fragaria* × *ananassa* Duch.) [48], peach (*Prunus persica* (L.) Batsch) [49], and apple (*Malus* × *domestica* Borkh.) [50].

The different results may be due to species-specific responses to the typology of mulching film and growing conditions, and/or other agricultural practices.

Furthermore, the soil texture affected muskmelon yield. In particular, the sandy loam soil elicited a 36.3% increase in yield compared with that obtained in clay–loam soil, as evidenced by the higher number and average weight of marketable fruits (+43.8% and +7.8%, respectively). Qualitative parameters were also positively affected by SL soil, with notable 14.1%, 10.1%, 22.8%, and 13.4% increases for polyphenols, carotenoids, antioxidant activity, and firmness, respectively. In terms of TSS content and dry matter percentage of fruits, the highest values were obtained on clay–loam soil, probably due the greater availability of certain micro-nutrients.

The combination CL-LDPE enhanced color parameters (brightness and hue angle) of muskmelon fruits, while the combination CL-MB produced the highest unmarketable yield, probably due to the direct contact of fruit with the soil (degradation of MB film under the weight of fruit), with consequent quality reduction accentuated by the higher water content in CL soil compared with SL.

It should be noted that early degradation of MB film occurred during fruit ripening; this behavior was previously reported by Filippi et al. [29] and Martin-Closas [5]. In both soils, during the fruit ripening, accelerated degradation of the biodegradable film was localized the areas under the developing melon fruits. Such degradation was observed associated with fruits weighing 400–500 g, with a frequency of nearly 100% (data not reported). It is thought that under the weight of the growing fruit, the contact between the mulching film and the soil was accentuated and the contact area became wet, promoting premature degradation of the film that in some cases stuck to the fruit peel and decreased the crop’s commercial value, but did not appear to promote fruit rot. The remaining area covered by the Mater-Bi^®^ film did not exhibit this kind of early degradation and stayed intact until the end of the crop cycle.

## 4. Materials and Methods

### 4.1. Experimental Design, Setting, and Crop Management

The experiment was conducted under plastic greenhouses in two private farms located on the Caserta plain; the first was the Ferrara farm located in Villa Literno (41°00′ N 14°04′ E, 10 m above sea level), and the second the Pellegrino farm in Giugliano, Campania (40°95′ N, 14°08′ E, 12 m asl).

The two farms were characterized by different soil textures. The Ferrara soil was clay–loam while the Pellegrino soil was sandy loam; their complete chemical and physical properties are reported in Table 5.

The experimental design involved a factorial combination with the typology of soil (clay–loam: CL, or sandy loam: SL) as the first factor, and the mulch film as the second factor. In particular, black biodegradable film based on Mater-Bi (MB), grade EF04P, width 1.6 m and thickness 15 microns, was compared with a black low-density polyethylene film (LDPE), width 1.6 m and thickness 30 microns.

Each treatment was replicated four times and each replicate corresponded to a single greenhouse. The tested crop was muskmelon, cv Pregiato (Clause), a netted melon hybrid with fruits characterized by long shelf life and highly resistant to *Fusarium oxysporum* f.sp. *melonis* races 0, 1, 2.

The films were applied by hand one week before the transplant, with plastic drip lines for irrigation concurrently rolled out under them. The transplants were made on 25 March 2015 in Ferrara farm, and 28 March 2015 in Pellegrino farm; the planting density was 5000 plants per hectare.

All agricultural practices on the farms were carried out as usual. In the first three weeks after transplant, short periods of irrigation (10–15 min) were alternated with fertigation supplying mainly nitrogen; thereafter, only fertigation was applied in order to provide simultaneously water and nutrients, mainly potassium. On average, farmers provided 50 kg N ha^−1^ and 100 kg K_2_O ha^−1^, and no phosphorus was added. Crop protection interventions were focused on control of aphids and oidium, and the choice of pesticides took into account the need to protect the bee hives introduced into the tunnels to increase flower pollination and fruit setting. No weed control was necessary, thanks to the good coverage of the soil by both mulching films.

### 4.2. Yield Measurements

In the Ferrara farm, five harvests took place starting on June 8 and continuing until June 24. In the Pellegrino farm, there were 11 harvests conducted from 12 to 28 June.

Under each greenhouse, the harvests were performed on a sampling area of 14 plants, corresponding to approximately 28 m^2^.

At each harvest, the number and weight of marketable and unmarketable fruits were determined; unmarketable fruits were those that were deformed, decaying, or weighed below 700 g.

To determine the dry matter percentage, a representative sample for each treatment and replicate was weighed and then oven dried at 70 °C until it reached a constant weight.

### 4.3. Temperatures Measurements

In both farms, the soil temperature was continuously monitored over two periods of the muskmelon cycle: from 30 to 45 days after transplant (DAT) and from 60 to 75 DAT. Two probes (Vantage Pro2, Davis Instruments) per each mulching film were placed at a soil depth of 10–15 cm.

### 4.4. Physical–Chemical Qualitative Traits and Color Parameters of Fruits

At the third harvest, the following organoleptic and nutritional qualities were determined by analyzing three fruits per replicate. The properties that were measured included color, firmness, pH, titratable acidity, total soluble solids, carotenoids, polyphenols, flavonoids, and antioxidant activity.

The qualitative determinations were made using fruit juice obtained by homogenizing small pieces of fruit in distilled water, and centrifuging at 15,000× *g* for 15 min.

Total soluble solid content (TSS expressed as °Brix) was measured using a digital refractometer (Sinergica Soluzioni, DBR35, Pescara, Italy).

Titratable acidity (TA) was determined by titrating 10 mL juice with 0.1 N NaOH, and the results expressed as g citric acid per liter of juice.

Flesh juice pH was measured using a digital pHmeter (Jenway, Staffordshire, UK).

Firmness was determined using a digital penetrometer (T.R. Turoni srl, Forlì, Italy) equipped with an 8 mm diameter probe; the measurements were taken from two opposite sides of three fruits per each replicate. Results were expressed in Newtons (N).

The Commission International de l’Eclairage (CIELAB) color parameters (L*, lightness, C, chroma, and hue angle (h)) were assessed using a colorimeter (CR5, Minolta Camera Co., Tokyo, Japan) applied to the fruit flesh obtained from fifteen fruits per treatment, according to the process reported by McGuire [51].

Bioactive compounds were extracted using methanol solution (80% *v*/*v*) according to the method described by Petriccione et al. [52]. Supernatants were filtered and then used for the various assays.

The Folin–Ciocalteu method was applied to determine the polyphenol content using 100 µL of methanol extract [53], and the results expressed as mg gallic acid equivalent (GAE) per 100 g fresh weight (FW).

Aluminum complex formation was assessed to evaluate flavonoid content [54], with results expressed as catechine equivalent (CAE) per 100 g of fresh weight.

Total carotenoids were extracted from flesh melon fruits using methanol (1:10 *w*/*v*) and analyzed spectrophotometrically at 470 nm according to the method reported by Wellburn [55]. Results were expressed as milligrams per 100 g fresh weight (FW).

Total antioxidant activity (AA) was measured using 1.1-diphenyl-2-picryl-hydrazil (DPPH) according to the method by Brand-Williams et al. [56]; results were expressed as µmol Trolox equivalents (TE)/g FW, according to a dose-response curve constructed using Trolox as standard.

### 4.5. Statistical Analysis

All data were subjected to 2-way analysis of variance (ANOVA) using the SPSS software package (SPSS version 22, Chicago, IL, USA). Experimental factors were Soil (S) (clay–loam and sandy loam) and Mulch (M) (Mater-Bi and PE). The means were separated using Tukey’s test at *p* ≤ 0.05.

## 5. Conclusions

The current study indicates that the use of biodegradable mulching films may be a valid alternative to traditional low-density polyethylene. Our results confirmed the capacity of the biodegradable film (MaterBi^®^) to increase muskmelon yield significantly, but the especially innovative and important aspect of this research is the improvement of fruit quality achieved with the use of biodegradable mulching. The MaterBi^®^ film greatly improved most of the qualitative parameters (flavonoids, polyphenols, antioxidant activity, and total soluble solids) that play key roles in guiding consumer choices. Meanwhile, current trends in consumer choice are for products that are environmentally friendly (biodegradable mulching films do not face the disposal problem affecting plastic film) and healthy (MaterBi^®^ increases bioactive compounds with antioxidant activity). Moreover, although we did not perform an economic evaluation, it is possible to highlight the convenience of using biodegradable films. Currently, the cost of these films is slightly higher than that of plastic films, but considering the total absence of disposal costs and the higher production that they elicit, we can certainly assume that biodegradable films represent a cost-effective approach for farmers.

Additionally, we observed better agronomical performance of muskmelon grown on sandy loam soil.

While these results are preliminary and require confirmation on a larger scale, using the same and other species under different growing conditions, they provide an interesting foundation for such research.

## Figures and Tables

**Table 1:** Effect of soil texture (clay–loam—CL; sandy loam—SL) and mulching (biodegradable film—MB; low density polyethylene—LDPE) on the number of marketable fruits and their average weight, unmarketable yield, and percentage incidence of unmarketable to marketable yield.

Treatments		Marketable Fruits (M)		Not Marketable (NM)	NM/M Yield
		*n°* *pt^−1^*	*g fruit^−1^*	*t ha^−1^*	*%*
Clay–loam	MB	4.1 ± 0.3	1134.7 ± 53.3	0.98 ± 0.10 a	3.73 ± 0.45 a
	LDPE	3.6 ± 0.1	1183.7 ± 8.6	0.43 ± 0.04 b	1.82 ± 0.17 b
Sandy loam	MB	5.6 ± 0.2	1272.8 ± 11.1	0.73 ± 0.04 b	2.08 ± 0.17 b
	LDPE	5.5 ± 0.1	1227.4 ± 10.5	0.61 ± 0.05 c	1.84 ± 0.15 b
CL		3.8 ± 0.2 b	1159.2 ± 26.7 b	0.70 ± 0.10	2.78 ± 0.39 a
SL		5.5 ± 0.1 a	1250.1 ± 10.5 a	0.67 ± 0.04	1.96 ± 0.11 b
MB		4.9 ± 0.3	1203.8 ± 34.5	0.85 ± 0.07 a	2.91 ± 0.35 a
LDPE		4.5 ± 0.3	1205.5± 9.7	0.52 ± 0.04 b	1.83 ± 0.11 b
**Significance**					
Soil texture (S)		**	**	ns	**
Mulching (M)		ns	ns	**	**
S × M		ns	ns	**	**

ns: not significant; **: significant at *p* ≤ 0.01. Different letters within each column indicate significant differences according to Tukey’s test (*p* ≤ 0.05). M = marketable; NM = not marketable.

**Table 2:** Effect of soil texture (clay–loam—CL; sandy loam—SL) and mulching (biodegradable film—MB; low density polyethylene—LDPE) on flavonoids, polyphenols, carotenoids, and total antioxidant activity of muskmelon fruits.

Treatments	Flavonoids	Polyphenols	Carotenoids	AA
	*mg CAE 100 g^−1^ fw*	*mg GAE 100 g^−1^ fw*	*µg 100 g^−1^ fw*	*µmol TE g^−1^ fw*
**Soil**				
Clay–loam	0.578 ± 0.02	26.9 ± 0.90 b	504.1 ± 14.6 b	1.84 ± 0.05 b
Sandy loam	0.567 ± 0.03	30.7 ± 0.98 a	554.7 ± 11.0 a	2.26 ± 0.12 a
**Mulching**				
MB	0.641 ± 0.03 a	31.7 ± 0.99 a	530.3 ± 8.4	2.28 ± 0.08 a
LDPE	0.504 ± 0.02 b	25.9 ± 0.68 b	528.5 ± 17.5	1.83 ± 0.08 b
**Significance**				
Soil texture (S)	ns	**	**	***
Mulching (M)	***	***	ns	***
S × M	ns	ns	ns	ns

ns: not significant; ** and ***: significant at *p* ≤ 0.01 and *p* ≤ 0.001, respectively. Different letters within each column indicate significant differences according to Tukey’s test (*p* ≤ 0.05). CAE = catechine equivalent; GAE = gallic acid equivalent; AA = antioxidant activity; TE = Trolox equivalent.

**Table 3:** Effect of soil texture (clay–loam—CL; sandy loam—SL) and mulching (biodegradable film—MB; low density polyethylene—LDPE) on pH, titratable acidity, total soluble solids, firmness, and dry matter percentage of muskmelon fruits.

Treatments	pH	Titratable Acidity	Total Soluble Solids	Firmness	DM
		*g citric acid L^−1^ juice*	*°Brix*	*N*	*%*
**Soil**					
Clay–loam	6.72 ± 0.03	2.08 ± 0.05	13.3 ± 0.26 a	23.8 ± 0.72 b	15.2 ± 0.28 a
Sandy loam	6.70 ± 0.03	2.09 ± 0.06	12.3 ± 0.20 b	27.0 ± 0.39 a	12.8 ± 0.13 b
**Mulching**					
MB	6.68 ± 0.03	2.12 ± 0.07	13.6 ± 0.18 a	24.9 ± 0.62	14.1 ± 0.30
LDPE	6.74 ± 0.03	2.05 ± 0.06	12.0 ± 0.22 b	25.9 ± 0.66	14.0 ± 0.32
**Significance**					
Soil texture (S)	ns	ns	**	**	***
Mulching (M)	ns	ns	***	ns	ns
S × M	ns	ns	ns	ns	ns

ns: not significant; ** and ***: significant at *p* ≤ 0.01, and *p* ≤ 0.001, respectively. Different letters within each column indicate significant differences according to Tukey’s test (*p* ≤ 0.05). DM = dry matter.

**Table 4:** Effect of interactions of soil texture (clay–loam—CL; sandy loam—SL) and mulching (biodegradable film—MB; low density polyethylene—LDPE) on color parameters L* (brightness), C (chroma), and h (hue angle) of muskmelon fruits.

Treatments		L*	C	h
Clay–loam	MB	53.9 ± 1.2 b	35.6 ± 1.4	61.6 ± 0.6 b
	LDPE	61.9 ± 1.0 a	41.6 ± 1.4	63.5 ± 0.4 a
Sandy loam	MB	55.4 ± 0.8 b	38.4 ± 0.8	61.2 ± 0.2 b
	LDPE	58.9 ± 0.5 b	44.5 ± 0.5	61.5 ± 0.2 b
CL		57.9 ± 1.1	38.6 ± 1.1 b	62.5 ± 0.4 a
SL		57.2 ± 0.6	41.4 ± 0.7 a	61.4 ± 0.1 b
MB		54.7 ± 0.7 b	37.0 ± 0.8 b	61.4 ± 0.3 b
LDPE		60.4 ± 0.6 a	43.0 ± 0.8 a	62.5 ± 0.3 a
**Significance**				
Soil texture (S)		ns	**	**
Mulching (M)		***	***	**
S × M		**	ns	*

ns: not significant; *, **, and ***: significant at *p* ≤ 0.05, *p* ≤ 0.01, and *p* ≤ 0.001, respectively. Different letters within each column indicate significant differences according to Tukey’s test (*p* ≤ 0.05).

**Table 5:** Chemical and physical properties of soil.

Parameters	Measure Unit	Ferrara	Pellegrino
**Texture**			
Sand	%	38.0	71.0
Silt	%	29.0	17.0
Clay	%	33.0	12.0
		Clay-Loam *	Sandy-Loam *
**N total** (Kjeldhal method)	%	0.097	0.108
**P_2_O_5_** (Olsen method)	ppm	67.6	96.6
**K_2_O**	ppm	2393.0	2135.0
**Magnesium**	ppm	255.0	119.0
**Calcium**	ppm	2760.0	2320.0
**Sodium**	ppm	257.0	322.0
**Organic matter** (Bichromate method)	%	1.9	1.6
**C/N**		11.3	8.6
**Active limestone**	% CaCO_3_	1.1	0.7
pH		7.7	7.3
**Electrical conductivity**	dS m^−1^	0.246	0.109

* USDA.

## Data Availability

The datasets generated for this study are available on request to the corresponding author.
